# Letter from Joel Hendricks, M. D.

**Published:** 1849-07

**Authors:** Joel E. Hendricks

**Affiliations:** Auburn Ind.


### ARTICLE IV.

### *Letter from Joel E. Hendricks, M. D.*

Messrs Editors:—

You have published an article (art. 5,) in the Journal for February and March, in which the writer, Dr. Jackson, expresses his disbelief in the agent called malaria, and assigns, as a cause of what are termed malarial fevers, a superabundance of carbon in the blood. I believe that few physicians, if any, will admit the positions taken in that article, yet I deem it not improper to make a few remarks on the arguments of the writer.

1st. Dr. Jackson supposes that, because there is in existence other agents than malaria sufficiently potent to produce disease, it is unphilosophical to attribute it to malaria, which he regards as an "unknown or doubtful cause."

2d. He supposes the cause of fevers, especially bilious, intermittent and remittent fevers, to be a superabundance of carbon in the blood.

3d. He supposes the reason why persons are more afflicted with ague in some portions of the country than in others, is because they are more subject to the influence of heat and cold, breathe more carbonic acid gas, and eat less food that will unite with carbon, and form new compounds.

1st. Respecting the existence of malaria, it is true that of its essential nature we know nothing, and respecting its origin we are uncertain; in these respects it may be said to be unknown, but, we infer its existence from its effects, as certainly as we infer the existence of a specific agent which produces typhus fever, measels, &c. The evidence in the several cases is the same.

2d. I do not pretend to deny the potency of the agents mentioned by Dr. Jackson, in producing disease, but I deny that we have any evidence of their being the principal agents in producing *ague* by causing a suppression of the perspiration. On the contrary universal experience teaches us that the general result of suppressed perspiration is catarrhal fever.

3d. To infer that fevers, especially bilious, intermittent, and remittent, are the result of irritation produced by a superabundance of carbon retained in the blood, because in those fevers carbon appears in excess, combined with the excretions, appears to me quite illogical, for it is well known that the functions of the skin especially, are performed vicariously by other organs. Hence, if the functions of the skin are disturbed by any cause, so as to suppress perspiration, we would be led to expect that the perspirable matter would appear in the other excretions; but we could not logically infer that this perspirable matter is in all cases the sole cause of the irritation. On the contrary we have reason to believe that in ague especially, perspirable matter is *not* the principle cause of the irritation, for, if it were, the abundant excretions should afford permanent relief.

It is probable therefore, that in bilious, intermittent, and remittent fevers, instead of a superabundance of carbon in the blood being the sole cause of the irritation, if a superabundance of carbon exist at any time during the fever, it is merely a consequence of the deranged action of the skin and glands generally, induced by the introduction into the system of the specific agent which we call malaria.

4th. As to the reasons assigned by Dr. Jackson why agues do not prevail in some localities while they do in others, it is sufficient to say, that all the advantages enumerated, and all other advantages relative to the amount of carbon in the blood are frequently posessed without exemption from the ague.

Yours very respectfully.

JOEL E. HENDRICKS

Auburn Ind., April 28th, 1849.

